# Evolving norms: social media data analysis on parks and greenspaces perception changes before and after the COVID 19 pandemic using a machine learning approach

**DOI:** 10.1038/s41598-022-17077-3

**Published:** 2022-08-02

**Authors:** Sohyun Park, Seungman Kim, Jaehoon Lee, Biyoung Heo

**Affiliations:** 1 Department of Plant Science & Landscape Architecture, University of Connecticut, Storrs, USA; 2 Department of Educational Psychology and Leadership, Texas Tech University, Lubbock, USA; 3 James Corner Field Operations, New York, USA

**Keywords:** Environmental social sciences, Health care

## Abstract

This study provides a novel approach to understand human perception changes in their experiences of and interactions with public greenspaces during the early months of COVID-19. Using social media data and machine learning techniques, the study delivers new understandings of how people began to feel differently about their experiences compared to pre-COVID times. The study illuminates a renewed appreciation of nature as well as an emerging but prominent pattern of emotional and spiritual experiences expressed through a social media platform. Given that most park and recreational studies have almost exclusively examined whether park use increased or decreased during the pandemic, this research provides meaningful implications beyond the simple extensional visit pattern and lends weight to the growing evidences on changing perceptions over and the positive psychological impacts of nature. The study highlights the preeminent roles parks and greenspaces play during the pandemic and guides a new direction in future park development to support more natural elements and nature-oriented experiences from which emotional and spiritual well-being outcomes can be drawn.

## Introduction

The world has faced the greatest global health crisis in the twenty-first century. As of March 2022, more than six million lives worldwide had been lost to the novel coronavirus (COVID-19), causing severe health problems. The consequences have touched every sector of society including public health and socio-economic instability with unprecedented changes in lives in a short time period, and these consequences are still continuing. The specific impacts of lockdown, face mask use, and social distancing were multifaceted at global scale. For example, Ribeiro et al.^1^ found that the COVID-19 lockdown has been associated with the deterioration of mental health, including post-traumatic stress disorder (PTSD), anger and anxiety. Similarly, Le et al.^2^ found that people with severe PTSD had significantly higher depression, anxiety, and stress levels among Vietnamese under the nationwide partial lockdown. Wang et al.^3^ compared two countries, one encouraged face masks (China) and the other that discouraged face masks (Poland) and discovered a higher level of anxiety and depression in Polish that reported social stigma associated face mask use. Tran et al.^4^ examined the impact of COVID-19 on economic well-being in Vietnam, where they reported 66.9% of the participants (N = 341) had household income loss due to the impact of pandemic during the national social distancing. Furthermore, research shows COVID-19 patients have a higher psychological impact of the outbreak (e.g., depression, anxiety and stress) than psychiatric patients and healthy populations^5^. A systematic review that examined the onset and frequency of depression in post-COVID-19 syndrome indicates that the frequency of depressive symptoms over the 12 weeks following SARS-CoV-2 infection ranged from 11 to 28%^6^.

Since a state of emergency was first declared in March 2020, the pandemic has recast our understanding of daily life spanning from the individual and household levels to institutional and societal levels. Primary administrative orders and public health guidance (e.g., stay-at-home and social distancing) have caused physical and psychological distress, fear, and depression^7–9^. As a result, many indoor civic spaces including indoor recreation facilities were unavailable to the public in the early pandemic and public outdoor spaces were some of the only spaces for people to have much-needed respite and peace from an overwhelmed sense of anxiety and affliction.

Since late 2020, a growing number of studies and reports have documented a new surge in the use of parks and greenspaces in response to the pandemic. The data have been primarily gathered from data monitoring programs and social surveys targeting outdoor recreation managers or park/trail users. For instance, the Connecticut Trail Census^10^ found a substantial spike in trail counts during the early months of the pandemic through a counter sensing system, with a general increase in the use of trails across the state. There was also a notable increase in the number of first-time rural trails users^11^ who might have sought safer spaces that may not have been present in their urban residences. Similar patterns have been documented in other parts of the country. For example, weekly bicycle and pedestrian use on local trails in Minnesota rose during this period compared to weather-adjusted averages (day-of-week, daily high temperature, and daily rainfall) to the extent that some popular trails were closed due to overcrowding^12^.

Along with the new trend of a sudden increase in local rural trail use, urban greenspaces in densely populated cities such as New York City remained popular destinations and were even more important for mental and physical health than before the pandemic^13^. As such, the COVID-19 pandemic has elevated the latitude of parks and everyday greenspaces from a personal recreational option to an essential amenity regardless of the location. In addition, the presence, accessibility, proximity, and safety of parks and greenspaces (as a general term and with regard to physical distancing practices) have become central subjects related to equity and justice issues in park research and discourse^14–16^. In addition to the recent findings regarding the changing pattern of park visitation, some studies have examined the changing perception on urban greenspaces and psychological outcomes related to park and nature exposure during the pandemic. For example, Olszewska-Guizzo et al.^17^ studied the neurophysiological response to urban spaces through brain scans in highly urbanized city of Singapore and suggested that high quality of greenspaces can be a key to mentally healthier cities. Ribeiro et al.^1^ claim that the exposure to nature was associated with better mental health outcomes during lockdowns and pointed out the natural features associated with improved mental health differed in two countries of interest (Portugal and Spain) where different restrictions and epidemiological situations occurred. Likewise, Olszewska-Guizzo et al.^18^ found that busy urban scenes induced a hemodynamic response associated with stress and anxiety, while urban greenspaces caused a significant and marginally significant decrease in average oxyhemoglobin (Oxy-Hb) in the visual cortex, suggesting green scenes can be an important factor to offset the negative neuropsychological impact during and post the pandemic.

As research related to the impact of COVID-19 pandemic on modified perception emerges, however, how human perceptual changes have made in response to people’s direct experiences of and interactions with parks and greenspaces is relatively less understood. This study aims to understand the changes in people’s cognitive experiences of parks and greenspaces before and after the COVID-19 pandemic using innovative machine learning techniques through a social media lens.

## Study settings

### Spatial setting

**Figure 1:**
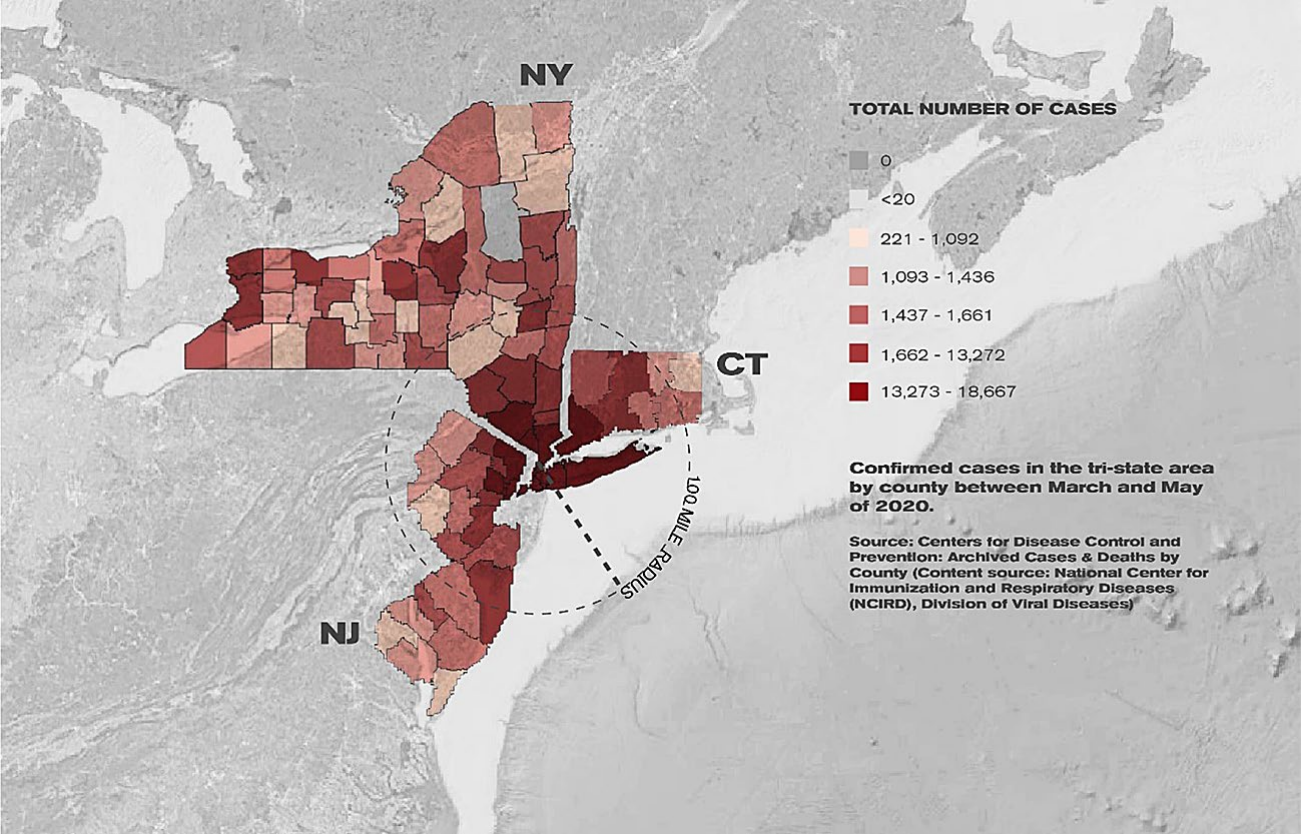
Confirmed cases in the tri-state area by county between March and May 2020. The color-coded map showing the total number of COVID-19 confirmed cases were obtained from the Centers for Disease Control and Prevention Covid Data Tracker site, in the Archived Cases & Deaths by County section. The background base map was downloaded from Google Earth Pro and modified in Adobe Photoshop to make gray color tone. Google Earth map and color-coded data map were put together in Adobe Illustrator.

The study area includes three adjoining northeastern U.S. states: New York, New Jersey, and Connecticut. The tri-state area experienced hot spots during the first wave of the coronavirus epidemic and then was a safe-to-travel region only in the autumn of 2020 before the second wave hit in the winter. The three states are closely connected with significant floating populations moving across state boundaries for jobs and regional economy, centered largely on New York City. New York City had an unprecedented and rapid explosion of COVID-19 hospitalizations, becoming the global epicenter of the outbreak in the early pandemic period (our study period), and had a significant impact on high caseloads in the neighboring counties in New Jersey and Connecticut^19^ as shown in Fig. 1.

### Temporal setting

Twitter data were collected for the two-month period immediately after the World Health Organization (WHO) declared that COVID-19 was a global pandemic. The specific time span for data collection ranged from March 13, 2020, through May 12, 2020. For comparison, the exact time period was applied to four preceding years (2016, 2017, 2018, and 2019) to obtain pre-COVID twitter data.

### Conceptual setting

Target spaces of interest included public parks, forests, trails, and other types of greenspaces, designed or natural. Social media users were engaged with these spaces as they remained open during the early pandemic months. Since the foci of this study were on physical green space features, as a natural form or for passive recreational purposes, we excluded spaces used mainly for outdoor social gathering (e.g., plazas, amphitheaters) from our study. Likewise, beaches were not included as access to these locations was restricted per administrative order from the three states during the time span of the analysis.

## Methods

### Data collection

This study analyzed Twitter data from a convenience sample of residents in the states of New York, New Jersey, and Connecticut. Twitter premium APIs were utilized along with predefined keywords to extract Twitter posts (tweets) relevant to park and greenspace experiences. The keywords were in three discrete categories: (1) place type, (2) user experience, and (3) geography. For example, the *place type* keywords included “park”, “trail”, and “nature”. The *user experience* keywords were those illustrating recreational activities typically observed in public greenspaces. Last, the *geography* keywords were the names of the three states in full and abbreviated forms, which set the geographic locations of the tweets to be retained. We scrapped tweets generated over a two-month window (March 13–May 12) in each year from 2016 to 2019, and then in 2020 for a comparison to the previous years. Through this process, a total of 73,315 tweets were initially retained.

### Preprocessing

Prior to analysis, the scraped tweets were preprocessed as follows. First, tweets that were nonsensible or too short (i.e., < 60 characters) were excluded. Second, people’s names were identified by cross-referencing them to the 151,671 common surnames found in the U.S. 2002 Census and they were removed from the tweets. Next, the NLTK library in Python 3.8^20^was used to filter out stop words such as pronouns, prepositions, and postpositions^21^. For topic modeling, a TF-IDF weighting method was employed to identify words that frequently appeared exclusively within a topic^22^. Although the word “park” appeared frequently, this word was not considered in the analysis, because it appeared across multiple topics and thus non-distinctive to a particular topic. Table 1 shows the number of tweets for each state and each year of interest after the preprocessing stage. A total of 41,861 tweets were ultimately accounted for in the analysis.

**Table 1:** The number of valid tweets included for data analysis for 2-month window (March 13–May 12) in each year from 2016 to 2020, and the number of total Twitter users.

Year	The number of extracted Tweets				The number of total Twitter users (millions)
	State			Total	
	NY	NJ	CT		
2016	3679	778	477	4934	318
2017	3350	825	446	4621	330
2018	5264	1250	686	7200	321
2019	7224	1894	926	10,044	330
2020	11,670	2442	950	15,062	353
Total	31,187	7189	3485	41,861	–

The data for the number of total Twitter users are from “backlinko.com”.

### Data analysis

**Figure 2:**
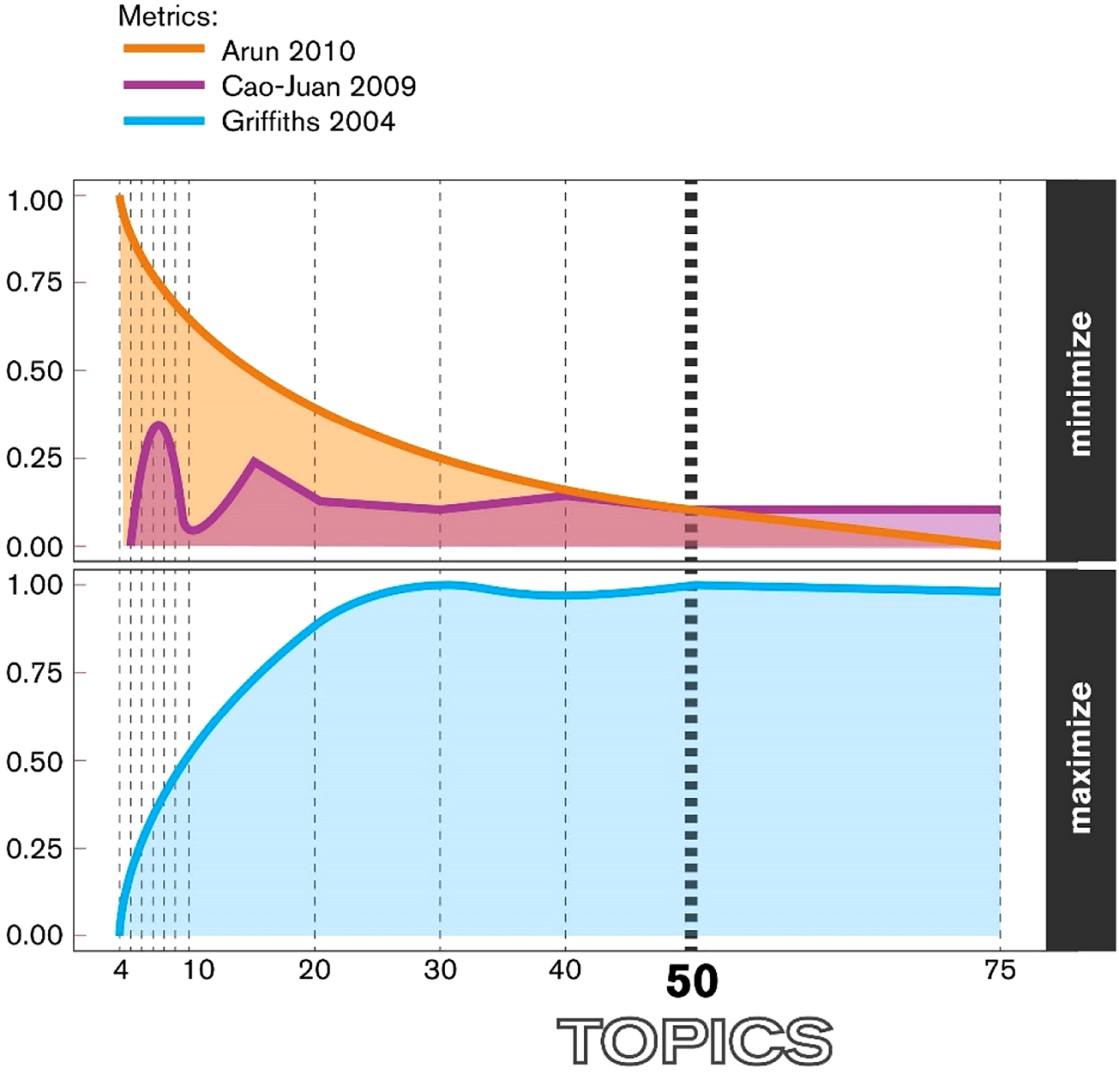
The optimal number of topics indicated by different metrics.

Data analysis proceeded in four stages to examine changes in public cognition (beliefs, opinions, attitudes) about parks and greenspaces after the COVID-19. First, Latent Dirichlet Allocation (LDA^23^) a topic modeling technique used to identify classes (groups) of words that represent key topics in a set of documents (e.g., tweets in our case), was performed using topic models^24^ and ldatuning^25^ packages in R^26^. In the initial stage of LDA, the optimal number of topics was determined^27^ by inspecting various metrics. Figure 2 shows the metrics of Griffiths^28^, Cao et al.^29^ and Arun^30^, all of which represent the method to arrive at the optimal number of topics. The optimal point was converged into where Griffith’s value was maximized (approaching 1) and Cao and Juan’s and Arun’s values were stabilized.

**Figure 3:**
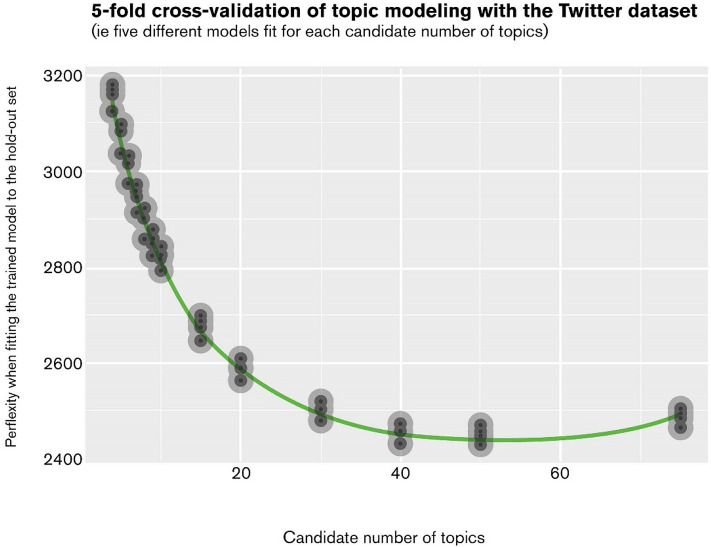
Perplexity in cross-validation.

The optimal number of 50 was confirmed by 5-fold cross-validation with perplexity^27,31^. In other words, the validation method used five different machine learning models to find the best fit for each candidate number of topics. A lower value of perplexity indicates better generalization performance of *p*, a predictive distribution over the words that were learned from a training dataset. As shown in Fig. 3, the perplexity value decreased and became the lowest (i.e., best fit) with 50 topics. After the number 50 was reached, the perplexity value increased indicating overfitting.

Sentiment analysis was then conducted using the TextBlob library in Python to extract the writers’ emotional states from the tweets. Specifically, for each tweet, polarity and subjectivity were calculated. Polarity ranges from − 1 (extremely negative mood) to + 1 (extremely positive mood), and the tweets were categorized as *positive* (polarity ≥ 0.30), *neutral* (− 0.30 < polarity < 0.30), or *negative* (polarity ≤  − 0.30). Subjectivity goes between 0 and 1. Tweets with a subjectivity value < 0.50 were classified as *objective*; otherwise as *subjective*.

We then conducted longitudinal cluster analysis, an unsupervised machine learning technique, on the 50 topics identified from the earlier LDA stage. This analysis classified the topics into a few clusters that shared unique joint trajectories over the 5-year period. Specifically, the changes in three factors (frequency of the topic, average polarity of the tweets, and average subjectivity) over time were calculated for each topic. Then, the topics that showed a similar pattern of changes (trajectories) were grouped together using the kml3d package in R^32–34^. Last, using SAS 9.4, a chi-square test and *t*-test were performed to compare the number and proportion of tweets as well as the polarity and subjectivity of the tweets before and after the pandemic, respectively.

## Results

### Latent Dirichlet allocation (topic modeling)

Of the 50 aggregated topics statistically grouped using LDA, qualitative cross-reference evaluation confirmed that only 20 topics were deemed interpretable and relevant to the study; the remaining 30 topics and data (words in these topics) were excluded from further analyses. Among the 20 topics, two topics and corresponding words therein (italicized in Table 2) overlapped with each other and thus merged into a single topic (Topic 4) leaving 19 topics for analysis. Table 2 shows the final selected 19 topics and the words that are most representative of these topics. The words under each topic were identified through machine-aided techniques based on the frequency and uniqueness (i.e., the same words appearing across the topics were excluded from the final selection). The topics were then labeled in a way that best characterizes the words as follows: negative feelings (Topic 1), mask-wearing (Topic 2), enjoying nice weather (Topic 3), hiking trail (Topic 4), baseball (Topic 5), breathing fresh air (Topic 6), love and thank nature and God (Topic 7), children playing outside (Topic 8), positive feeling (Topic 9), staying at home (Topic 10), social distancing (Topic 11), enjoying and celebrating a beautiful day (Topic 12), water bodies (Topic 13), pets (dogs) (Topic 14), love watching and hearing nature (Topic 15), running (Topic 16), health (Topic 17), driving/parking cars (Topic 18), and biking (Topic 19).

**Table 2:** Important words for the 19 final topics.

Topic 1	Topic 2	Topic 3	Topic 4	Topic 5	Topic 6	Topic 7	Topic 8	Topic 9	Topic 10
Really Shit Lol Ass Fucking Fuck Crazy	Many People Mask Without Wearing Face Virus	Nature Weather Nice Spring Enjoy Cold Warm	Trail Hiking Hike Trails Appalachian Miles Rail	Run Home Hit Game Ball Inning Baseball	Get Air Fresh Need Nature Outside Breath	Nature Life Love Thank God World True	Kids School Play Outside Children Young Playground	Like Feel Love Better Much Outside Feeling	People Stay Home Live Safe Staying Inside

Topic 11	Topic 12	Topic 4	Topic 13	Topic 14	Topic 15	Topic 16	Topic 17	Topic 18	Topic 19
Social Distancing Space People Distance Safe Socially	Day Beautiful Happy Enjoy Celebrate Perfect Gorgeous	Trail Hiking National Mountain Shoes Boots Trip	Beach Lake Island Preserve Falls Pond Creek	Dog Cute Leash Pet Animals Wildlife Owner	Nature Trees Birds Love Flowers Watching Sounds	Run Mile Race Runners Track Road Marathon	Health Help Outdoors Mental Stress Physical Energy	Car Right Lot Parking Drive Street Waiting	Biking Ride Lane Protected Sidewalk Safety Cycling

**Figure 4:**
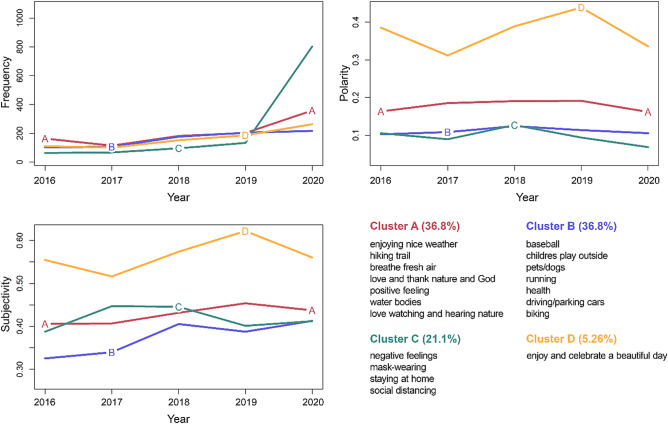
Four clusters representing unique trajectories of frequency, polarity, and subjectivity.

The longitudinal cluster analysis that investigated the trajectories of the 19 topics relative to their frequency, polarity, and subjectivity over the 5 years (2016–2020) resulted in four unique trajectories (clusters). Cluster A (36.8%) included Topic 3 (enjoying nice weather), Topic 4 (hiking trail), Topic 6 (breathing fresh air), Topic 7 (love and thank nature and God), Topic 9 (positive feeling), Topic 13 (water bodies), and Topic 15 (love watching and hearing nature). The polarity and subjectivity of this cluster remained the same or slightly increased over the years, but the frequency increased considerably after the pandemic. Cluster B (36.8%) was characterized as having low and consistent frequency, polarity, and subjectivity. This cluster included Topic 5 (baseball), Topic 8 (children playing outside), Topic 14 (pets/dogs), Topic 16 (running), Topic 17 (health), Topic 18 (driving/parking cars), and Topic 19 (biking). Cluster C (21.1%) included Topic 1 (negative feelings), Topic 2 (mask-wearing), Topic 10 (staying at home), and Topic 11 (social distancing). This cluster demonstrated a huge increase in frequency and a slight drop in polarity after the pandemic. However, the subjectivity has not changed significantly over time. Finally, Cluster D (5.26%) included only one topic (Topic 12: enjoying and celebrating a beautiful day), and this cluster showed the highest polarity and subjectivity compared to other clusters, which had significantly declined after the pandemic—i.e., more negative and objective tweets compared to the previous years (see Fig. 4).

**Figure 5:**
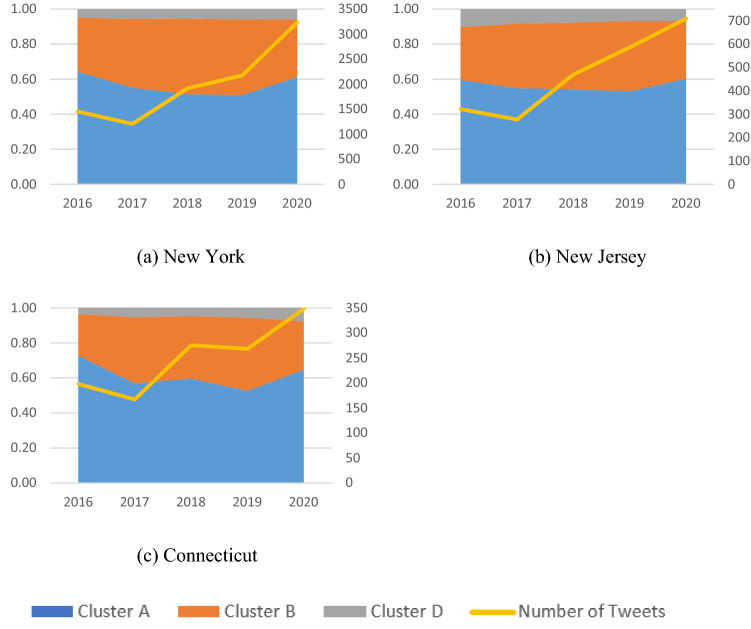
Changes in the proportion of clusters and the total number of tweets over the 5 years (2016–2020). The left and right vertical axes represent the proportion of clusters and the number of tweets, respectively.

Note that approximately 70% of the Cluster C tweets were generated after the COVID-19 pandemic. Excluding this cluster, the number and proportion of tweets in other clusters were further compared before and after the pandemic. Table 3 shows the number of tweets in Clusters A, B, and D from 2016 to 2020 for the three states (NY, NJ, CT) combined and individually. Although there was a shortfall in 2017, the overall number of tweets in these clusters more than doubled from 2016 (1974 tweets) to 2020 (4287 tweets). The results of the chi-square test indicated that the proportions of tweets across different clusters (i.e., relative size) also significantly changed over the years (*χ*^2^(3) = 2063.31, *p* < 0.0001). More specifically, the proportion of Cluster A tweets continued to decrease before the pandemic (from 64 to 51%), but in 2020 it sharply increased (to 61%), returning to the 2016 level. In contrast, the proportion of Cluster B tweets gradually increased before the pandemic (from 30 to 42%) and then declined to 32% in 2020. Cluster D remained in the same proportion (6%) throughout the period. As Fig. 5 illustrates, the increase in the number of tweets was greatest in New York (222% increase), followed by New Jersey (220%) and Connecticut (176%). In particular, the increase in the last 2 years (i.e., right before and right after the pandemic) was remarkable in New York (148%), followed by Connecticut (130%) and New Jersey (121%). The proportions of tweets across Clusters A, B, and D and the changes over time were similar among the three states.

**Table 3:** The number of tweets from 2016 to 2020 by state and cluster.

		2016	2017	2018	2019	2020
Total	Cluster A	1271 (64%)	911 (55%)	1404 (53%)	1559 (51%)	2631 (61%)
	Cluster B	593 (30%)	637 (39%)	1104 (42%)	1285 (42%)	1393 (32%)
	Cluster D	148 (6%)	163 (6%)	220 (6%)	273 (6%)	732 (6%)
NY	Cluster A	935 (64%)	664 (55%)	985 (51%)	1107(51%)	1978(61%)
	Cluster B	449 (31%)	472 (39%)	827 (43%)	938 (43%)	1064 (33%)
	Cluster D	70 (5%)	69 (6%)	103 (5%)	131 (6%)	188 (6%)
NJ	Cluster A	192 (60%)	152 (55%)	255 (54%)	311 (53%)	428 (60%)
	Cluster B	97 (30%)	102 (37%)	179 (38%)	235 (40%)	233 (33%)
	Cluster D	33 (10%)	23 (8%)	36 (8%)	40 (7%)	48 (7%)
CT	Cluster A	144 (73%)	95 (57%)	164 (60%)	141 (53%)	225 (65%)
	Cluster B	47 (24%)	63 (38%)	98 (36%)	112 (42%)	96 (28%)
	Cluster D	7 (4%)	9 (5%)	13 (5%)	15 (6%)	27 (8%)
	Total	1974	1649	2660	3030	4287

### Semantic analysis

The polarity and subjectivity of the tweets are presented in Table 4. A paired-samples *t*-test was conducted to examine the changes in the polarity and subjectivity of the tweets before and after the pandemic (2016–2019 vs. 2020). Overall, the polarity significantly decreased (*t* = 8.29, *p* < 0.001), but it was still in the “neutral” range. Interestingly, unlike other clusters, the tweets in Cluster B showed an increased polarity, albeit not statistically significant. The subjectivity significantly moved in the direction of “subjective” (*t* =  − 5.49, *p* < 0.001), which was marked in Clusters A and B but not the other two clusters. All three states showed an increase in subjectivity, but it was significant only in New Jersey (Table 4).

**Table 4:** Descriptive statistics and t-test results on polarity and subjectivity.

	Polarity			Subjectivity		
	Before	After	*t*	Before	After	*t*
Cluster A	0.18 (0.27)	0.16 (0.24)	2.41*	0.41 (0.30)	0.44 (0.23)	− 3.54***
Cluster B	0.10 (0.05)	0.11 (0.23)	− 1.47	0.36 (0.00)	0.41 (0.24)	− 6.79***
Cluster C	0.09 (0.27)	0.07 (0.23)	3.24**	0.42 (0.28)	0.41 (0.22)	1.27
Cluster D	0.39 (0.29)	0.34 (0.25)	2.69**	0.58 (0.29)	0.56 (0.24)	0.75
New York	0.17 (0.29)	0.17 (0.24)	0.21	0.41 (0.29)	0.43 (0.24)	− 1.42
New Jersey	0.14 (0.27)	0.12 (0.24)	6.14***	0.40 (0.29)	0.43 (0.23)	− 5.91***
Connecticut	0.17 (0.28)	0.11 (0.24)	6.27***	0.42 (0.29)	0.42 (0.24)	− 0.37

*p < 0.05, **p < 0.01, ***p < 0.001.

## Discussion and conclusion

### “Tweeting” more about nature and spiritual experiences during the COVID-19 pandemic

The results indicate that the pandemic has transformed how people perceive parks and greenspaces in the eastern tri-state region during the initial surge of the pandemic. The model-driven topic classification method and longitudinal clustering outcomes turned out to be legitimate to understand the domains of human interests related to parks and greenspace experiences. Unsurprisingly, the topics associated with COVID-19 safety measures (e.g., mask wearing, staying at home, and social distancing) showed a significant increase in tweet data to the extent that they were clustered into a discrete category (Cluster C). It is also understandable that traditional park activities (e.g., ball-playing, biking/running, dog walking) were still prominent topics on social media, as represented in Cluster B. The fact that there were no dramatic changes in people’s traditional park use patterns between pre- and post-COVID tweets reflects that people still may have found value in what they used to do in parks and greenspaces, and these recreational activities might have been perceived as safe enough when practiced alone or with immediate family.

Interestingly, people’s interests noticeably shifted to natural components after the pandemic. This change is likely tied to the positive sentimental attachment to parks and greenspaces, as identified in Clusters A and D. For example, “feeling fresh air” appeared to be a critical value in people’s experiences during the first shock of the pandemic. In fact, some passive experiences such as bird watching or listening to birds and water sounds, were not very common in pre-COVID tweets but became important experiences as the pandemic ramped up. More importantly, emotional and spiritual expressions elicited from people’s interaction with nature (e.g., loving and thanking nature and God) appeared to be an emerging yet strong theme in the post-COVID tweets in each state and across the study region. These results are aligned with previous findings in public health research that have clinically proven the benefits of nature during the pandemic. Part of the research include horticultural therapy where nature reduced plasma IL-6 level significantly and has shown the potential to prevent inflammatory disorders and hematopoietic support^35^. Nature was also proved to have effects on stronger frontal alpha asymmetry (FAA) values, which is commonly associated with the approach-related motivation and positive emotions^36^. In addition to the nature’s horticultural effects, Ng et al.^37^ highlight the critical roles of social connectedness as a social determinant of health (e.g., ameliorating COVID-19 triggered massive inflammation), which implies the importance of public parks as a both natural and social space.

The findings of this study infer that people became more attentive to the natural features and conditions than before the pandemic, began to link nature exposure to sentimental and spiritual well-being, and reappreciated the natural and health values of public greenspaces compared to their typical recreational and aesthetic aspects. Although this study cannot claim a direct correlation between the motivation of spiritual well-being and park visitation (Irvine et al.^38^ for relevant research), the benefits of emotional and spiritual well-being, anticipated or unanticipated, likely impacted the positive park experiences and outcomes. This finding suggests that the benefits of public greenspaces can be expanded to a dimension of spirituality where people experience self-reflection, introspection, meditation, and other inner feelings^39^. These feelings are known to be benefits of distant wilderness areas, but they have not often been associated with public greenspaces at the local level.

The results might have been influenced by the general increase in the number of tweets pertaining to parks and greenspaces in 2020, which, in itself, is inspiring, as it denotes emergent public interest in parks and greenspaces at the personal communication level. Nevertheless, people’s renewed appreciation of nature and immediate linkage to emotional and metaphysical realms illuminate the preeminent roles that parks and greenspaces play during the pandemic. The overall mood for the entire set of tweet data, however, moved toward a more negative tone, which is presumably due to the severity of the pandemic rather than being related to park visits and experiences.

### Advantages and disadvantages of using social media data

As social media data have become increasingly abundant, they are being utilized as a primary data source in many human–environment interaction studies to investigate broader patterns of human interest and experiences^40,41^. The greatest advantage of using social media data is that it can minimize subject response bias because respondents are not aware of the intent of the study^42^. In other words, the gathered data are a collection of spontaneous responses from test subjects, which can later be utilized as input for analysis. However, in a typical social survey, respondents may have a prior perception of the survey intent as they are informed by and cognizant of the goals of the research before participating in the survey. In addition, survey instruments can only take responses at a specific point in time and may not reflect the actual perception trends over a longer timeframe. Another strength of the social media approach is the ability to obtain a comprehensive (literally all) set of data that fulfills certain conditions that the research defines (in our case, all Twitter posts that contain preset keywords) during a desired period of time. In contrast, it is often a challenge for social surveys to secure enough samples. A known caveat of the social media approach, however, is that the sample data are limited to users of social media and do not capture potential respondents who are not on social media platforms. In addition, provided that tweets are voluntary expressions of personal feelings and instant thoughts, the language of the Twitter posts are only assumed to represent social media users’ individual cognition and inner ideas about their experiences. Nonetheless, it serves as a valid approach to address time-sensitive phenomena such as the pandemic from a vast amount of data that are relatively readily available and have been formed in a voluntary and instantaneous manner.

Under unique circumstances such as COVID-19, data collection is especially bounded by time and sampling. As random sampling is often not viable, researchers tend to rely on convenience sampling for easy and timely access to target subjects. In fact, many recent studies have capitalized on this type of method by adding COVID-19-related questions to existing surveys or by creating a quick questionnaire to sample a population that is easy to contact or reach, causing a generalization issue.

### Summary and future research

This study attempted to understand people’s perceptual changes in their interactions with parks and greenspaces during the early months of COVID-19 using social media data combined with a machine-learning approach. We categorized key words from tweet data into 19 topics to understand what new parameters people began to appreciate in parks and greenspaces compared to pre-COVID times. The results demonstrate that people were more inclined to appreciate natural experiences in these spaces and tended to link them to their emotional and spiritual domains, as reflected in these very common themes across the three states in this study. Albeit geographically fractional, the research revealed that there is a clear difference between the early COVID-impacted timeframe versus non-impacted years in that people perceived nature in public greenspaces as a substantial emotional and spiritual lift. Most park and recreational studies have almost exclusively examined whether park use increased or decreased during the pandemic, but this research focused more on whether and how park user experiences were felt differently beyond the simple extensional visit pattern. The study demonstrates a new pattern of more nature-oriented activities in public greenspaces and how natural components have been important in people’s emotional and spiritual experiences during the COVID-19 pandemic. Although the focus of the study was on identifying the general pattern from the nature-related tweeter texts, more biological and scientific research would be needed to better understand the mechanism how park exposure benefits the mental, emotional, and spiritual health.

The implications of this research include the emergent and adaptive roles of public greenspaces, in response to changing human needs in disruptive phenomena. The study suggests a deliberate incorporation of biophysical characteristics of wilderness settings (e.g., elevations, water, naturalness, unique scenery and landscapes) into our public park systems to facilitate the emotional and spiritual dimensions of our experiences (e.g., feeling unity with nature and unity with oneself) in addition to typical park experiences^43^. We also argue that we need to position nature as a vital element in contemporary public park designs and development decisions.

One limitation of this study is that we relied largely on an indirect method to understand people’s nuanced feelings, senses, ideas, and perceptions expressed through a social media platform. Therefore, the results only represent the general pattern of public perceptions rather than direct reflection from individuals. Given that we continue to struggle with another surge in COVID-19 cases driven by the highly transmissible Delta and Omicron variants, we are likely to experience continued pandemic concerns, a new wave of restrictions on public gathering, and entry into a “with-COVID” era. Future research needs to verify if the findings remain true in post-Covid period as tweeter data gets accumulated. Future research also needs to understand the genuine desire of the public for parks and greenspaces as an essential and equitable resource in current and future health crises. The research agenda should fill the gaps between park provision and people’s new motivation to be safe in outdoor spaces during the pandemic. We plan to expand our analysis to the entire country in an elongated timeframe to trace the extended trends over time.

## Data Availability

The data that support the findings of this study are available from Twitter, but restrictions apply to the availability of these data, which were used under Twitter Policy for the current study, and so are not publicly available. Data are however available from the authors upon reasonable request and with permission of Twitter. In this case, the data request can be made to the corresponding author at sohyun.park@uconn.edu.
